# Photosynthetic capacity and pigment distribution of a siphonous green alga, *Dichotomosiphon tuberosus*

**DOI:** 10.1007/s11120-025-01148-3

**Published:** 2025-05-21

**Authors:** Soichiro Seki, Koichi Kobayashi, Ritsuko Fujii

**Affiliations:** 1 Graduate School of Science, Osaka City University, 3-3-138, Sugimoto, Sumiyoshi, Osaka 558-8585 Japan; 2 Graduate School of Science, Osaka Metropolitan University, 1-1 Gakuencho, Naka-ku, Sakai, Osaka 599-8531 Japan; 3 Research Center for Artificial Photosynthesis, Osaka Metropolitan University, 3-3-138 Sugimoto, Sumiyoshi-ku, Osaka, 558-8585 Japan; 4 Present Address: Institute for Protein Research, Osaka University, 3-2, Yamadaoka, Suita, Osaka 565-0871 Japan

**Keywords:** *Dichotomosiphon tuberosus*, Non-photochemical quenching, Light-harvesting complex, Siphonaxanthin, Siphonein, Siphonous green algae

## Abstract

*Dichotomosiphon tuberosus* is one of the Bryopsidales, a siphonous, unicellular multinucleate ulvophyte. Bryopsidales typically occur in the ocean and contain unique carbonyl carotenoids siphonaxanthin (Sx) and its ester siphonein (Sn) in their major light-harvesting pigment-protein complexes, allowing them to utilize the green light available in the deep ocean for photosynthesis. However, unlike other Bryopsidales, *D. tuberosus* occurs in fresh water and is reported to contain Sn but not Sx. *D. tuberosus* inhabits deep lakes around the world, but in Okinawa, Japan, it inhabits very shallow waterways. Here, we measured the photosynthetic capacity of *D. tuberosus* collected from Okinawa waterway and compared it with another intertidal Bryopsidale *Codium fragile*. *D. tuberosus* had higher photosynthetic electron transport capacity and stronger non-photochemical quenching than *C. fragile*, consistent with the brighter growth environments for *D. tuberosus* than *C. fragile*. We also measured the absorption spectra and the pigment compositions within the photosynthetic pigment-protein complexes from *D. tuberosus.* Green light absorption of each complex in *D. tuberosus* was weaker than that in *C. fragile.* In contrast, Chl *b* absorption in LHCII was stronger in *D. tuberosus* than in *C. fragile*, whereas the opposite was true in photosystems. This implies that a large proportion of the irradiated energy is absorbed by LHCII complex and quenched more efficiently. Our results indicate that the photosynthetic capacity of *D. tuberosus* is further optimized for higher light environments compared with *C. fragile*.

**Supplementary Information:**

The online version contains supplementary material available at 10.1007/s11120-025-01148-3.

## Introduction

*Dichotomosiphon tuberosus* grows in freshwater but is genetically classified as a member of the Bryopsidales (class Ulva), a primary group of marine macrogreen algae usually called siphonous green algae (Kleinig 1969). The siphonous green algae exhibit characteristic single and multinucleate “siphonous” giant cell structures (Kleinig 1969; Maekawa PCP 1986). Recent phylogenic analysis using multi-locus datasets confirmed that *D. tuberosus* is monophyly of the family Dichotomosiphonaceae (Cremen 2019). The phylogenetic analysis also suggested that *D. tuberosus* may be a freshwater survivor of a common ancestor with its genetic sister species showing preferences for saltwater (Curtis et al. 2008; Verbruggen 2009; Cremen 2019).

*D. tuberosus* was first discovered in Switzerland and has been found in Europe, North Africa, Pakistan, India, Myanmar, China, Japan, and North America for decades (Kishimoto 2023; Moestrup 1973). *D. tuberosus* tends to inhabit the bottom of freshwater lakes at depths of 4–6 m or more and in fields with unconsolidated substrates of sandy-silt mixtures, suggesting that the attenuated sunlight plays an important role in its growth (Neil 1985). However, in Okinawa prefecture, Japan, *D. tuberosus* occurs naturally in shallow waterways from abundant spring water (Yokohama 1989). It has been noted that the core Bryopsidales lack the functional xanthophyll cycle, which is a major mechanism for suppressing excess energy in a wide range of oxygenic photosynthetic organisms (Christa 2017). Despite the unique phylogenetic location and habitat preferences of *D. tuberosus* described above, its photosynthetic capacity remains unclear. Then, a question arises as to how the photosynthetic response of *D. tuberosus* differs from that of typical Bryopsidales.

As another characteristic, Bryopsidales accumulate unique carbonyl carotenoids, siphonaxanthin (Sx) with its ester siphonein (Sn) in a various composition. Accumulation of Sx and Sn in the antenna complex associates with the ability to thrive in the low-intensity green light available on the deep seafloor. In intertidal siphonous green algae, both Sx and Sn are bound to the photosynthetic antenna, absorbing green light and transferring the excitation energy to the photosystem II (PSII) to drive photosynthetic electron transport (Yokohama 1989). Those pigments are found bound to the major light-harvesting complex II (LHCII) (Anderson 1983) in the binding place of non-carbonyl carotenoid lutein (Lu) in the LHCII of vascular plants. Sx is biosynthesized from Lu, and Sn is biosynthesized from Sx. Sn is a fatty acid ester of the hydroxymethyl group (19-OH group) of Sx (Seki 2022b). Both Sx and Sn exhibit absorption bands around 450 nm in organic solvents, but they are responsible for the absorption band around 540 nm when bound to the antenna proteins (Anderson 1983). The high-resolution structure of LHCII from intertidal siphonous green algae, *Codium fragile* (Seki 2022a) and *Bryopsis corticulans* (Li 2023), confirmed that Sx and Sn bind to the lutein-binding sites, usually called L2 and L1 sites, respectively, and that only Sx accepts two hydrogen bonds from amino acid residues. The structural characteristics of Sx potentially generate the green light absorption of LHCII unique to marine algae (Gisriel 2023).

In the case of *D. tuberosus*, the contribution of the green light absorption band to photosynthetic function was confirmed by the fluorescence excitation spectrum and the action spectrum of oxygen evolution (Yokohama 1989). However, unlike other members of Bryopsidales, *D. tuberosus* contains Sn but not Sx (Kleinig 1969; Yokohama 1989). According to the above structure-based hypothesis, the presence of the green light absorption band in the Sx-free species is contradictory. From another perspective, it has been pointed out that in the LHCII, the lifetime of excited chlorophyll is shortened depending on the hydrophobicity of the binding xanthophylls (Ruban and Johnson 2010). As the polarity of Sx and Sn is significantly different, the xanthophyll composition of LHCII in *D. tuberosus* may contribute to the quenching function. Then, the second question arises as to how the pigment composition of each major photosynthetic apparatus of *D. tuberosus* differs from that of typical Bryopsidales. To clarify these points, it is necessary to characterize the photosynthetic apparatus of *D. tuberosus*, which has not yet been isolated and characterized.

Here, to evaluate the photosynthetic capacities of *D. tuberosus*, we performed pulse amplitude modulation (PAM) chlorophyll-*a* fluorescence analysis and compared the photosynthetic parameters in *D. tuberosus* with those in *C. fragile*. We also measured the pigment composition and absorption spectra of isolated photosynthetic complexes of *D. tuberosus* and compared them with those of *C. fragile.* Based on the photosynthetic capacity and photosynthetic machinery, we discussed the unique adaptive mechanisms that distinguish *D. tuberosus* from other Bryopsidales.

## Results

### The living environment of *D. tuberosus* in Okinawa prefecture

**Fig. 1:**
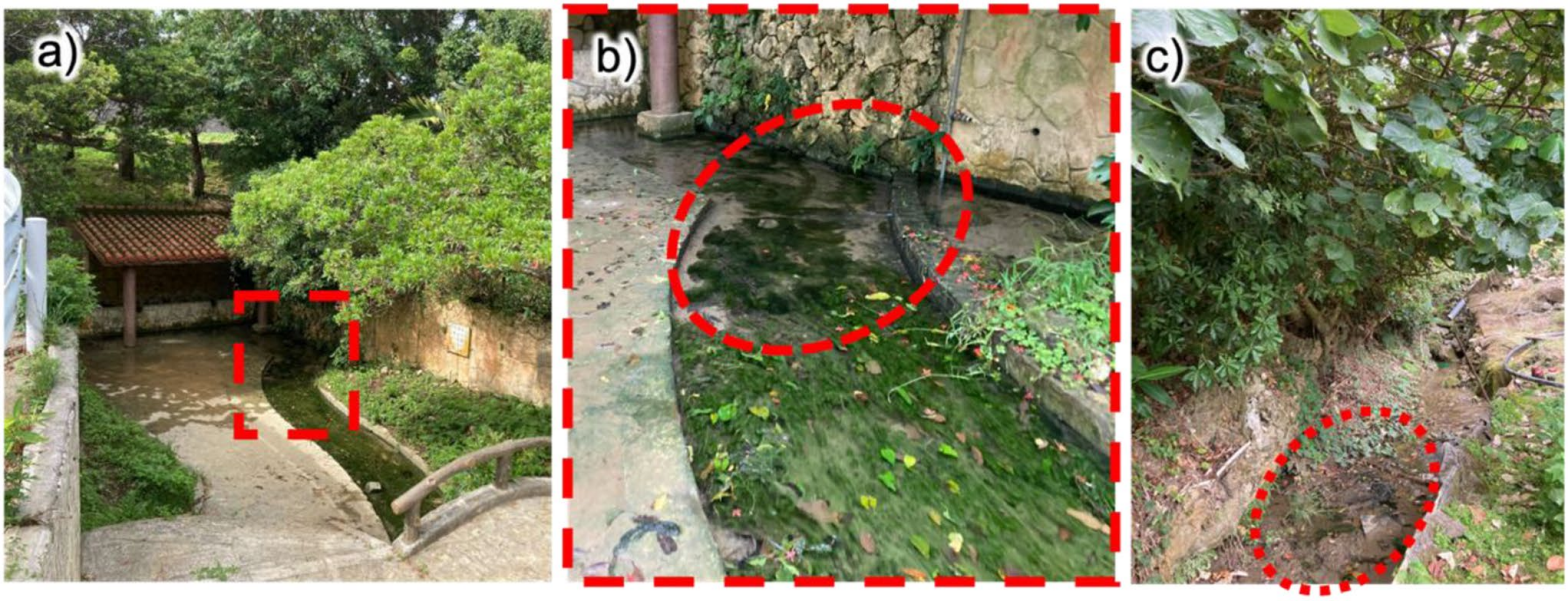
The habitat of *D. tuberosus*. **a**) Oyakebaru-ukka in Okinawa and **b**) its enlarged view, **c**) water cress field in Okinawa. Red circles indicate colony of this alga

In Okinawa prefecture, *D. tuberosus* mainly occurs near springs where fresh water gushes out or in running water areas where spring water flows, but it also grows in rivers and rice fields, considered as sunny locations (Yokohama 1989). *D. tuberosus* was harvested from a waterway called “Oyakebaru-Ukka” and an irrigation stream in a watercress field in the southern part of the main island of Okinawa (Fig. 1). In the former location, *D. tuberosus* adhered to the sand and clay on the bottom of the stream, forming a thick mat at water depths of less than 20 cm. Even within the same waterway, *D. tuberosus* formed almost pure colonies in areas shaded by roofs and trees (as indicated by a red dashed circle in Fig. 1b), while the mixtures of other green algae grew in clumps in other areas exposed to sunlight all day long (as shown in the foreground of Fig. 1b). In the latter watercress field, *D. tuberosus* also flourished in areas shaded by bushes (as indicated by a red dashed circle in Fig. 1c). These observations indicate that this alga grows advantageously in areas shaded by trees, bushes, and roofs. The light intensity was measured at Oyakebaru-Ukka. On this cloudy day in April, it was 300–500 photosynthetic photon flux density (PPFD, µmol photons m^− 2^ s^− 1^ at 400–700 nm region) at the water surface in this algae mat, while it was 700–1200 PPFD in a place without shade.

### Morphological identification of *D. tuberosus* using optical microscope

**Fig. 2:**
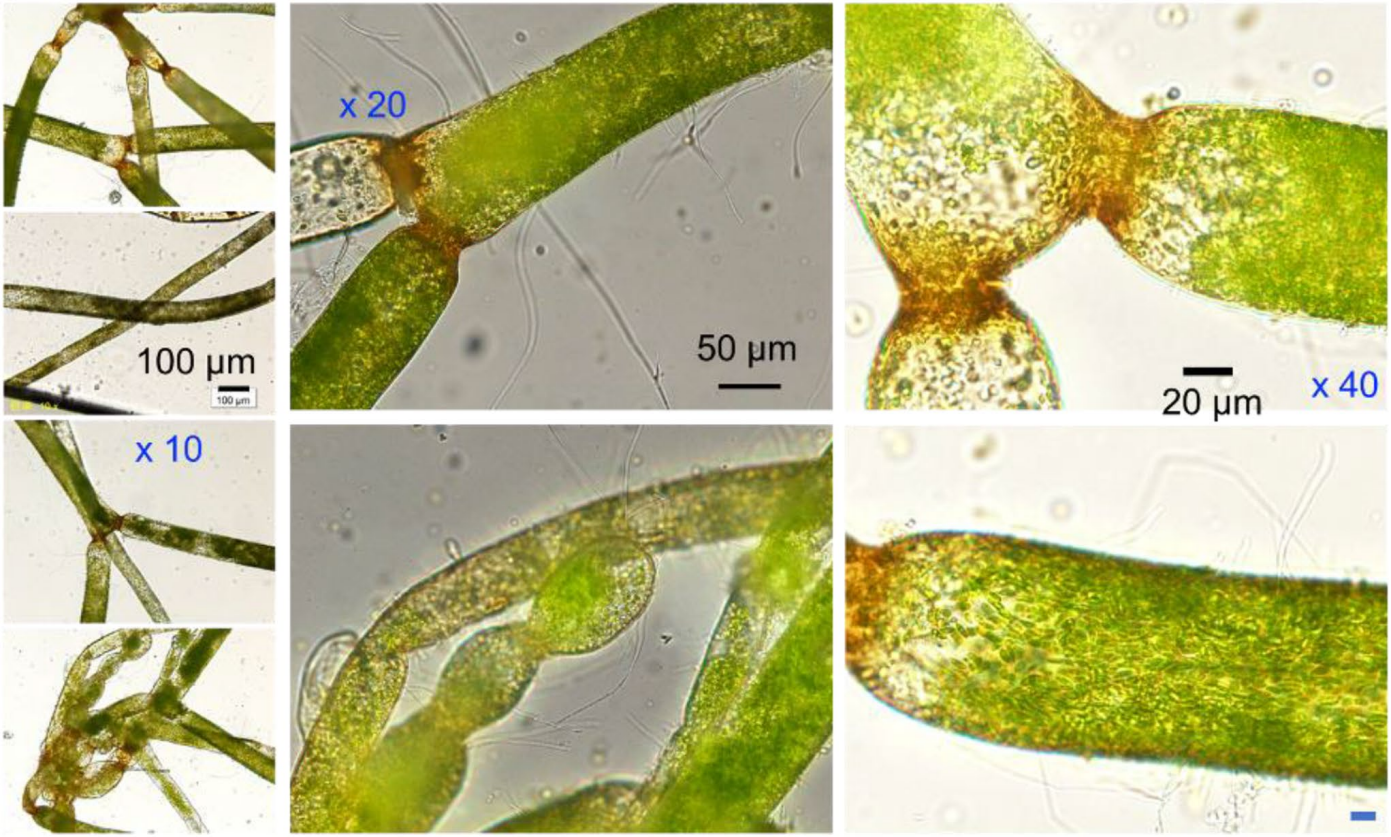
The micrograph images of *D. tuberosus*. Scale bars for (**a**-**d**), (**e**, **f**), (**g**,**h**) are indicated in **b**), **e**), **g**), respectively. Characteristic nods with brownish color (**a**, **c**, **d**, **e**, **g**, **h**), and characteristic unicell structure (**b**) were identified

*D. tuberosus* was identified using the characteristic micrographic feature (Neil 1985) of a “siphonous” tubular giant single-cell approximately 50 μm in diameter with dichotomous branches (split into two prongs) and constrictions at points along the length (Fig. 2a-h). The cells were normally filled by green chloroplasts in various densities and brown colored at the node possibly due to the accumulation of iron (Neil 1985). Interestingly, *D. tuberosus* found in Okinawa prefecture, unlike one found in Lake Simcoe, Ontario (Neil 1985), does not exhibit akinetes (Kishimoto 2023).

### Light-dependent photosynthetic capacity of *D. tuberosus* and *C. fragile*

**Fig. 3:**
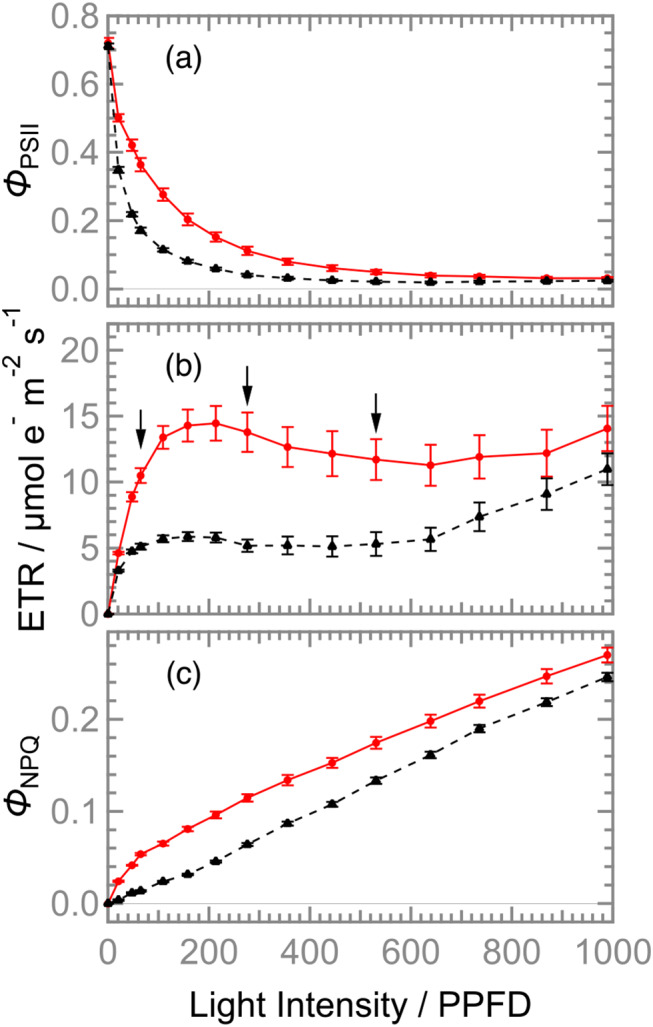
Dependence of actinic-light intensity on (**a**) *Φ*_PSII_, (**b**) ETR, and (**c**) *Φ*_NPQ_ in *D. tuberosus* (red solid lines) and *C. fragile* (black broken lines). Values and error bars indicate means ± SE (*N* = 4). ETR, electron transport rate, in µmol electron m^− 2^ s^− 1^; *Φ*_PSII_, the effective quantum yield of photosystem II; *Φ*_NPQ_, the quantum yield of non-photochemical quenching; PPFD, photosynthetic photon flux density in µmol photon m^− 2^ s^− 1^. Arrows indicate selected light conditions at low, moderate, and high light intensities, i.e., 65, 276, and 531 PPFD, respectively (See text)

To evaluate the photosynthetic ability of *D. tuberosus* under various light intensities, the maximum quantum yield of PSII (*F*_v_/*F*_m_) and light-intensity dependence of the effective quantum yield of PSII (*Φ*_PSII_), electron transport rate (ETR), and the yield of regulated energy dissipation (*Φ*_NPQ_) in *D. tuberosus* (red lines in Fig. 3) were compared with those in *C. fragile* (black broken lines in Fig. 3). The *F*_v_/*F*_m_ levels in *D. tuberosus* and *C. fragile* were 0.72 ± 0.02 and 0.71 ± 0.02, respectively, which are within the range of values for other ulvophycean algae grown under low light intensity (25 PPFD) (F_v_/F_m_ = 0.70–0.77), but slightly higher than those grown under high light (200 PPFD) (F_v_/F_m_ = 0.57–0.68) (Christa et al. 2017). Although a rapid attenuation of *Φ*_PSII_ was observed both in *D. tuberosus* and *C. fragile* as the actinic light intensity increased, *D. tuberosus* showed higher *Φ*_PSII_ levels than *C. fragile* particularly between 50 and 500 PPFD, resulting in higher ETR in *D. tuberosus* than *C. fragile*. In *D. tuberosus*, ETR reached its maximum value at 200 PPFD and retained the level with increased light intensity, whereas that in *C. fragile* had a small peak at 150 PPFD and gradually increased under strong light of 600 PPFD or more. The data suggest that the suitable light intensity for *D. tuberosus*, which is approximately 200 PPFD, is higher than that for *C. fragile*, which is 100–150 PPFD, although both species can maintain photosynthetic activity under stronger light intensities. In both *D. tuberosus* and *C. fragile*, the levels of *Φ*_NPQ_ gradually increased as the light intensity increased. *D. tuberosus* showed higher increasing rates of *Φ*_NPQ_ between 20 and 100 PPFD and higher *Φ*_NPQ_ levels under all light intensities than *C. fragile*. The data indicates that *D. tuberosus* has a higher thermal-dissipating ability of excess light energy than *C. fragile*.

### Induction and relaxation kinetics of photosynthetic parameters in *D. tuberosus* and *C. fragile*

**Fig. 4:**
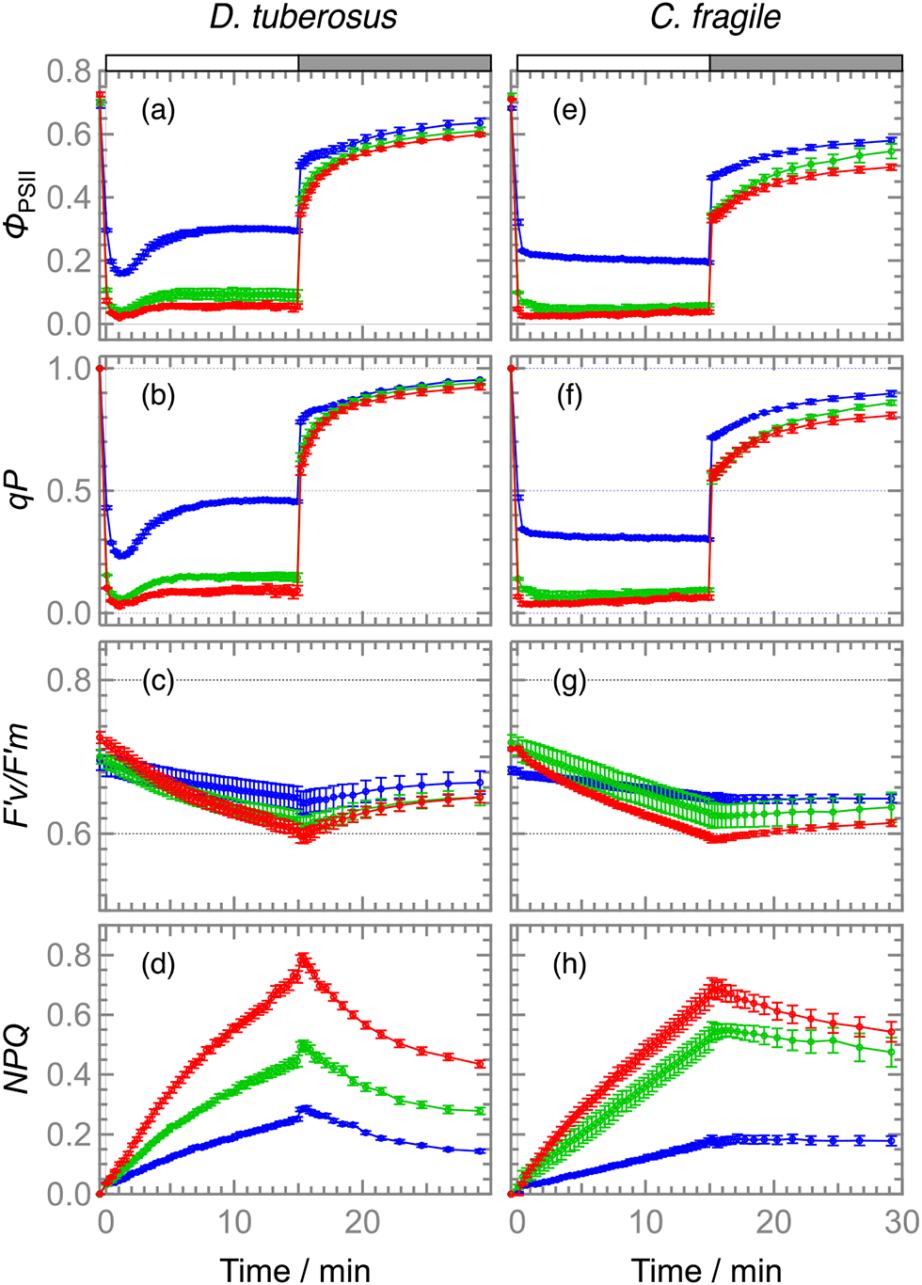
Induction and relaxation time course of photosynthetic parameters in *D. tuberosus* (left side panels) and *C. fragile* (right hand panels) upon irradiated by low (blue), moderate (green) and high (red) lights for 15 min (see Fig. 3 and text). (**a** and **e**) *Φ*_PSII_, (**b** and **f**) *q*_P_, (**c** and **g**) F’v/F’m, and (d and h) *NPQ*. Values and error bars indicate means ± SE (*N* = 4). F’v/F’m, the maximum quantum efficiency of open PSII; *NPQ*, the non-photochemical quenching parameters; *Φ*_PSII_, the effective quantum yield of photosystem II; *q*_P_, the coefficient of photochemical quenching

To gain further insight into the photosynthetic characteristics of *D. tuberosus* in comparison with *C. fragile*, the kinetics of the several photosynthetic parameters were determined during 15-min actinic irradiation at different intensities, followed by dark incubation for 15 min (Fig. 4). Based on the light response curves of the ETR (Fig. 3b), three representative light intensities, namely 65, 276, and 531 PPFD, were selected as low light (LL), medium light (ML), and high light (HL) conditions, respectively. In both *D. tuberosus* and *C. fragile*, the *Φ*_PSII_ levels were relatively high under 65 PPFD but were substantially decreased when the light intensities were increased to 276 and 531 PPFD, consistent with the data in Fig. 3a. *Φ*_PSII_ can be viewed as a product of two components, the coefficient of photochemical quenching (*q*_P_), which reflects the oxidation state of the primary electron acceptor plastoquinone Q_A_, and the maximum quantum efficiency of open PSII (*F′*_*v*_/*F′*_*m*_) under actinic light. In both *D. tuberosus* and *C. fragile*, *q*_P_ fluctuated similarly to *Φ*_PSII_ during measurement and strongly decreased in response to increased light intensity, whereas *F′*_*v*_/*F′*_*m*_ was relatively stable, suggesting that the redox state of the plastoquinone pool but not the PSII functionality is the limiting factor of the PSII photochemical efficiency particularly under strong light in both species. Of note, under all three light intensities, *D. tuberosus* showed a downward peak of *Φ*_PSII_ during the first several minutes of actinic irradiation with a gradual recovery, which was not observed in *C. fragile*. A similar fluctuation was also observed in *q*_P_ of *D. tuberosus* but not *C. fragile*, suggesting that excess reduction of the plastoquinone pool transiently occurred with actinic irradiation in *D. tuberosus*. The analysis of the non-photochemical quenching parameter (*NPQ*) showed that, in both species under all light conditions, the *NPQ* levels gradually increased during actinic irradiation and were not immediately relaxed after turning off the light. The data indicate that *D. tuberosus* as well as *C. fragile* do not develop the energy-dependent type of *NPQ* (*q*_E_), which is the fastest component of *NPQ* (Derks et al. 2015), regardless of the irradiated light intensities. Nevertheless, *D. tuberosus* showed faster relaxation of *NPQ* than *C. fragile* during dark treatment, so the *NPQ* mechanism working during actinic irradiation may be partially different between these two species. Although qE-type *NPQ* is associated with the xanthophyll cycle, no functional xanthophyll-cycle was observed in many species of Bryopsidales, including *Codium* sp. (Christa 2017). As *D. tuberosus* accumulates a certain amount of Vx, the xanthophyll-cycle was further evaluated by comparing the pigment composition before (control) and after 30 min of irradiation with white LED at 300, 700, and 1000 PPFD (Supporting information Table S1). However, no light-dependent decrease in Vx accumulation was observed, suggesting the absence of a functional xanthophyll cycle.

### Absorption spectra of isolated thylakoid proteins of *D. tuberosus*

**Fig. 5:**
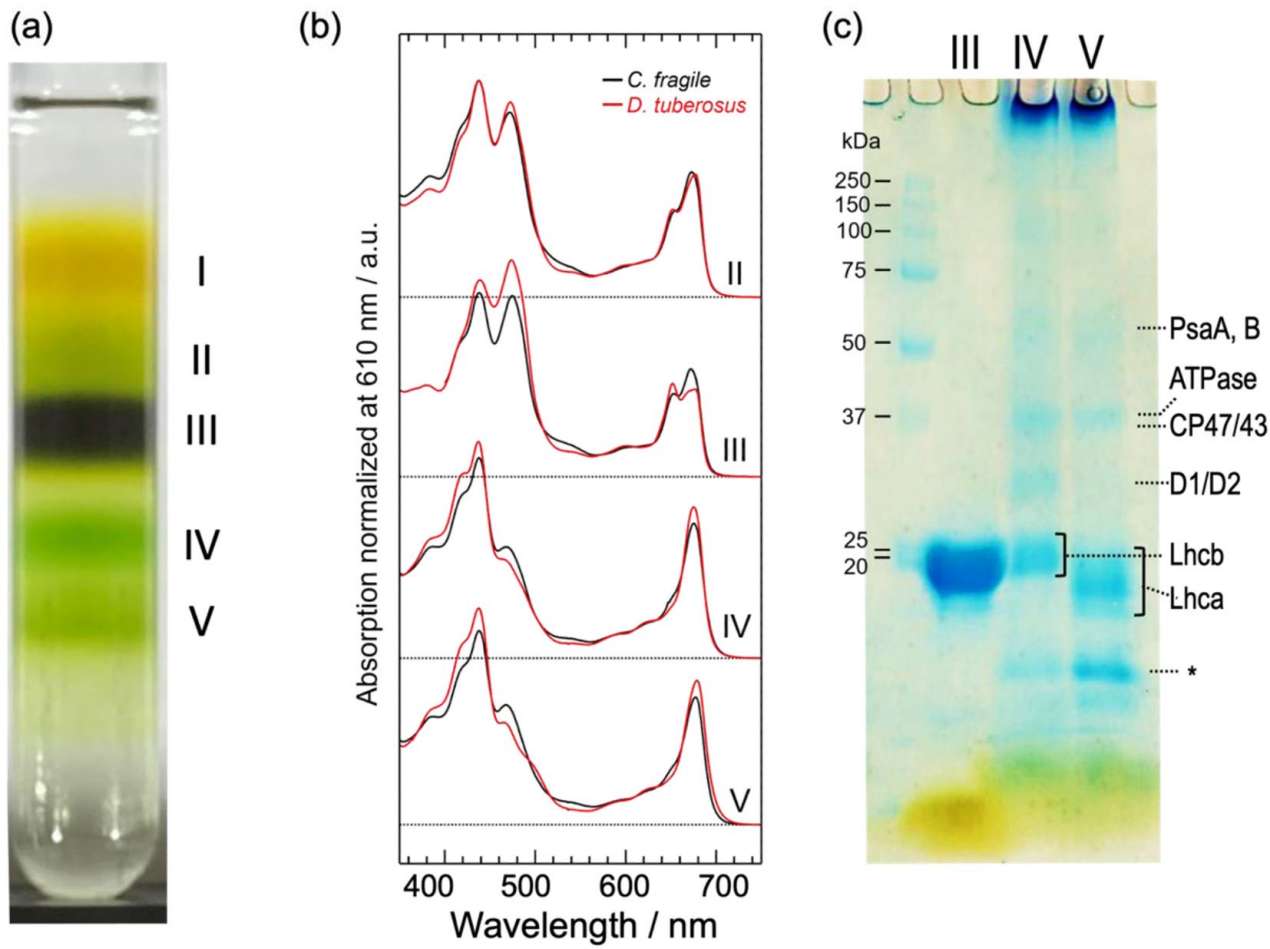
Thylakoid proteins from *D. tuberosus.* (**a**) The result of sucrose density gradient (SDG) using thylakoid membrane solubilized by 1% β-DDM. (**b**) The absorption spectra of fractions IIーV (red lines) with those of C. fragile (black line) at the same condition, and (**c**) the result of SDS-PAGE of SDG fractions III, IV, and V. An asterisk (*) indicates an unidentified band associated with the PSI core (see text)

The major process of photoprotective non-photochemical quenching involves specific carotenoids bound to the photosynthetic pigment-protein complexes, especially for LHCII (Ruban and Saccon 2022). To evaluate the relevance of the photosynthetic capacity and the unique pigment composition, the thylakoid proteins were isolated using sucrose density gradient (SDG) centrifugation. The resultant five fractions are shown in Fig. 5a. The separation pattern and relative concentrations of each fraction closely reproduce results for the intertidal siphonous green algae, such as *Bryopsis corticulans* (Wang 2013; Qin 2015) and *C. fragile* (Seki 2022b), and the dominant fraction (Fraction III) is likely to be trimeric LHCII. Those fractions are empirically assigned as follows: I, free pigments; II, a mixture of monomeric LHCs; III, LHCII trimer; IV, PSII and PSI cores; and V, PSI-LHCI supercomplexes. Fractions III, IV, and V were further identified using absorption spectra (Fig. 5b) and SDS-PAGE (Fig. 5c).

The absorption spectra of fractions II, III, IV, and V (Fig. 5b, red lines) exhibited typical characteristics following the above tentative assignments. Fractions II and III exhibit prominent absorption peaks at ~ 680 and ~ 440 nm due to Q_y_ and Soret transitions of Chl *a*, respectively, as well as comparable magnitude absorption peaks at 652 and 474 nm due to Chl *b*. In contrast, fractions IV and V have only minor contributions from the latter. This is consistent with the fact that in higher plants (Croce 2018) and green algae (Shen 2019; Quiniou 2015; Huang 2021; Qin 2019), Chl *b* is primarily located in antenna complexes. Fractions IV and V appear to have similar spectral shapes, but the Q_y_ peak maxima were 675 and 679 nm, respectively, and a broad plateau between 450 and 520 nm was better separated in fraction V. These spectral features resemble the absorption spectra of PSII-LHCII (Shen 2019) and PSI-LHCI (Huang 2021) of a green alga *Chlamydomonas reinhardtii*, respectively. Thus, fractions IV and V contain mainly PSII core and PSI-LHCI, respectively. The results of SDS-PAGE (Fig. 5c), empirically assigned according to the references (Shen 2019; Huang 2021), supported the above assignments. Fraction III shows a broad band of Lhcb (apoprotein of LHCII), consistent with the assignment of trimeric LHCII. The presence of the D1/D2 (and CP26/29) band only in fraction IV suggests that this fraction contains PSII. Fraction IV also contains a faint band assigned to PsaA/B and a relatively strong band below Lhca which is assignable to an unidentified protein associated with the PSI core (Barera 2012), suggesting that fraction IV may also contain PSI core. Fraction IV also contains a significant amount of Lhcb (LHCII). The presence of Lhca (apoprotein of LHCI) only in fraction V indicates that this fraction contains LHCI. In fraction V, the faint band below PsaA/B can be assigned to ATPase (Barera 2012), together with the absence of D1/D2 band, it is considered that there is almost no contamination with PSII.

The absorption spectrum of each fraction in *D. tuberosus* was further compared with those in *C. fragile* prepared at the same conditions (Fig. 5b, black lines). The absorption in the green region from 530 to 560 nm in each fraction is weaker in *D. tuberosus* than in *C. fragile*. However, fraction III of *D. tuberosus* exhibits a small but evident absorption peaking around 540 nm. This should correspond to the green absorption of the in vivo absorption spectrum of this alga, which had been suggested to be attributed to Sn in vivo in thalli of this alga (Yokohama 1989). This green absorption in fraction III in *D. tuberosus* exhibits a reduced bandwidth and a more distinct peak position compared with that of *C. fragile*. The Chl absorption spectral profiles of fraction IV and V differ between *D. tuberosus* and *C. fragile*. In fractions IV and V, both the Q_y_ and Soret absorption bands of Chl *b* are smaller in *D. tuberosus* than in *C. fragile*, whereas in fraction III, they are larger in *D. tuberosus* than in *C. fragile*. Fraction III in *D. tuberosus* exhibits a strong Q_y_ absorption of Chl *b* at 652 nm, the magnitude of which is comparable to that of Chl *a*, and a notably broad Q_y_ absorption of Chl *a* around 680 nm, which appears to be split into two peaks (670 and 677 nm). The absorption bands in the Q_y_ region reflect the excitation energies specific to Chl at each binding site of LHCII and thus are deeply correlated with the energy transfer pathway and efficiency. Therefore, LHCII of *D. tuberosus* is expected to have Chl energetics that differ substantially from those of the LHCIIs of other species studied so far.

### Pigment distribution of the major photosynthetic apparatus in *D. tuberosus*

**Fig. 6:**
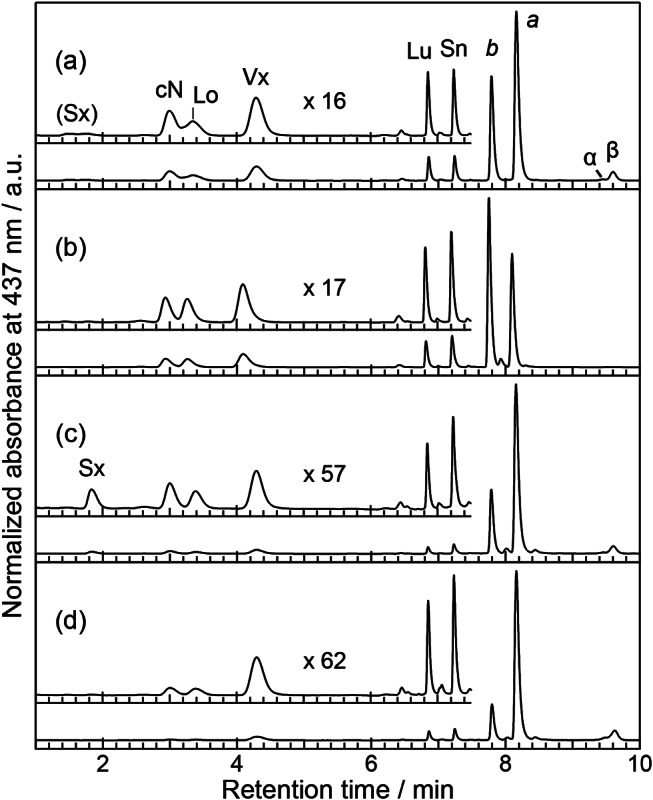
HPLC chromatograms of the pigments extracted from the algal body (**a**), and SDG fractions III (**b**), IV (**c**), and V (**d**), respectively. Each inset is a magnification of the xanthophyll area normalized by Vx, with the respective magnifications indicated in the figure. Abbreviations: *a*, chlorophyll *a*; *b*, chlorophyll *b*; β, β-carotene; cN, 9’-*cis*-neoxanthin; Lo, loroxanthin; Lu, lutein; Sn, siphonein; Sx, siphonaxanthin; Vx, violaxanthin

The pigment compositions of the algal body and the three fractions (III, IV, and V) of *D. tuberosus* were determined by a high-performance liquid chromatography (HPLC) equipped with a photodiode array detector (Fig. 6, Table S2) and compared with those of *C. fragile* (Table S3). As reported previously using thin layer chromatography (Yokohama 1989), the algal body of *D. tuberosus* contained four xanthophylls [9’-*cis* neoxanthin (cN), violaxanthin (Vx), lutein (Lu), and Sn] and Chl *b* and *a*, and two carotenes [α-carotene (α-Car), and β-carotene (β-Car)] in the order of elution. The high separation capacity of this HPLC system enabled the detection of loroxanthin (Lo). In addition, the algal body contained a trace amount of Sx as a broad peak (Fig. 6, Table S2). Notably, unlike *C. fragile*, where Sx was present in each fraction (Table S3), in *D. tuberosus* Sx was only detectable in fraction IV (PSII and PSI cores). Fraction III contains all xanthophylls except for Sx but no carotenes. In contrast, fractions IV and V (PSI-LHCI) contain both xanthophylls and carotenes, although the latter have a much lower relative number of total carotenoids to Chl molecules. Additionally, fraction V (PSI-LHCI) contains a smaller number of cN and Lo than fraction IV (PSII and PSI core). The Chl *a*/*b* ratio (Table S2) of the algal body was 1.58 ± 0.02, which is larger than that of *C. fragile* (1.04, Table S3). The values changed in the order of fraction III (0.59 ± 0.00), fraction IV (2.50 ± 0.06), and fraction V (4.80 ± 0.04). The Chl *a*/*b* ratio (0.60) of fraction III corresponding to LHCII was smaller than *C. fragile* LHCII (0.79) and other LHCII from land plants (~ 1.33), indicating *D. tuberosus* LHCII contains more Chl *b* than others.

In land plants, cN is only bound to Lhcb proteins at a proportion of one molecule per protein except for CP24 (Croce 2018). The Lhcb proteins constitute various LHCs responsible for energy transfer to PSII. These include trimeric LHCII and monomeric antennas (Croce 2018). Trimeric LHCII has four carotenoid binding sites per monomer, commonly referred to as L1, L2, N1, and V1 sites, whereas monomeric LHC only has the first three (Croce 2018). This is also true for a green alga *Chlamydomonas reinhardtii* (Huang 2021) and a siphonous green alga *Bryopsis corticulans* (Qin 2019). Therefore, cN can be considered an indicator of the presence of Lhcb protein. The fact that fraction V hardly contains cN is consistent with our assignment of fraction V to be PSI-LHCI which contains no Lhcb protein.

The relative amounts of xanthophylls to cN (Xant/cN ratio) calculated from Table S2 are listed in Table 1. The Xant/cN ratio of fraction III indicates that LHCII contains one Lo molecule, two Vx molecules, one Lu molecule, and one Sn molecule per monomer. The Xant/cN ratio of fraction IV showed that Vx and Sn are present in a similar number as cN per Lhcb protein, but Lo, Lu, and Sx are present in approximately half the number of cN.

**Table 1:** Xanthophyll composition relative to cN^*1^ (mol / mol cN) ^*2^

	Sx	Lo	Vx	Lu	Sn
Fraction III	0.03 ± 0.00	1.17 ± 0.02	1.90 ± 0.02	1.09 ± 0.01	1.26 ± 0.01
Fraction IV	0.48 ± 0.09	0.67 ± 0.07	1.27 ± 0.25	0.61 ± 0.10	0.86 ± 0.15

^*1^Abbreviation follows Fig. 6. ^*2^Values are calculated from Table S1

## Discussion

### Sunscreen effect due to sand attached to the surface

The light intensity suitable for bare *D. tuberosus* after thorough cleaning was found to be up to 200 PPFD (Fig. 3), while the ambient light conditions of its habitat in Okinawa were 300–500 PPFD or higher (Fig. 1). Notably, in natural conditions, the surface of the *D. tuberosus* thallus was covered with many foreign substances such as sand, clay, and mud from the channel bottom, giving it a whitish color. This implies that the irradiated light is highly scattered at the surface and the remaining light reaching to the chloroplasts is significantly attenuated. *D. tuberosus* is known to prefer to develop on sandy substrates (Neil 1985), and this preference may have led to the acquisition of scattering filters and ultimately contributed to its survival in this environment. It requires further investigation about the ability of adsorbing sand and/or mud.

### High photosynthetic capacity of *D. tuberosus* compared with *C. fragile*

The PAM-chlorophyll *a* fluorescence analysis revealed that of *D. tuberosus* has higher photosynthetic electron transport activity than *C. fragile* particularly between 100 and 500 PPFD. This observation is consistent with the light environments they inhabit; *D. tuberosus* flourishes in a waterway with relatively high light intensities (300–500 PPFD) (Fig. 1), whereas *C. fragile* thrives under low light intensities (30–90 PPFD) and higher light intensities above 150 PPFD decreases its growth rate (Hanisak 1979). The differences in *Φ*_PSII_ and ETR between these two species were mainly attributed to the higher *q*_P_ in *D. tuberosus* than *C. fragile*, indicating that *D. tuberosus* has a higher capacity to oxidize the plastoquinone pool under middle light intensity than *C. fragile*. Of note, *D. tuberosus* showed a gradual increase in *q*_P_ after a transient decrease during first several minutes after actinic irradiation (Fig. 4). Thus, these species may have a mechanism that promotes the oxidation of the plastoquinone pool rapidly after the strong reduction by a sudden light exposure. Many factors are associated with the oxidizing capacity of the plastoquinone pool, including every component of the electron transport chain downstream of PSII and the reductive metabolism such as the CO_2_ fixation, and future studies are required for addressing underlying mechanisms.

Another noticeable difference in photosynthetic characteristics observed between *D. tuberosus* and *C. fragile* is the stronger *Φ*_NPQ_ induction in *D. tuberosus* than *C. fragile* in response to increased actinic light intensities (Fig. 3). The data suggests the higher capacity of thermal energy dissipation in *D. tuberosus* than *C. fragile*, consistent with the blighter growth conditions for *D. tuberosus* in Okinawa. LHCII from *D. tuberosus*, one of the major non-photochemical quenching sites, shows the higher chlorophyll *b* content, drastically different Qy absorption pattern, and carotenoid composition compared with LHCII from other Bryopsidales (Seki 2022b; Li 2023). These unique characteristics may be associated with the thermal energy dissipation observed in *D. tuberosus*. Nonetheless, *D. tuberosus* and *C. fragile* showed no *q*_E_-type non-photochemical quenching (Fig. 4), characterized by fast development and relaxation of *NPQ* in response to light irradiation and subsequent dark treatment, respectively. Christa et al. (2017) compared the kinetics of *NPQ* among a wide range of ulvophycean algae and concluded that the macroalgae in the order Bryopsidales lack *q*_E_. They showed that those species have no functional xanthophyll-cycle contributing to the photoprotective non-photochemical quenching. *C. fragile* accumulates little Vx under dim light, but increases its accumulation under strong light (Seki 2022b). However, light-dependent composition changes among Vx/Ax/Zx were not detected (Christa 2017). Preliminary analysis of *D. tuberosus* showed a decrease in Vx after 30 min of exposure to intense white LEDs at 300 and 700 PPFD, but no corresponding increase in Ax or Zx was observed (Table S1). This suggests that *D. tuberosus* doesn’t have xanthophyll-cycle, as seen in several ulvophycean algae (Christa 2017). Iha et al. (2021) compared the genomes of *Ostreobium* and *Caulerpa* and showed that both PsbS and LHCSR genes, which are central factors of *q*_E_, were missing, suggesting that all Bryopsidales lack both genes. These data support our findings that *D. tuberosus* does not develop *q*_E_ regardless of the intensities of the irradiated actinic light. However, it should be noted that, in green algae, *q*_E_ is not constitutive and is established after acclimation to high-light conditions (Derks 2015). Therefore, we cannot exclude that pre-exposure to certain conditions is required to induce *q*_E_ in *D. tuberosus*. Despite the absence of the *q*_E_ mechanism, *D. tuberosus* showed faster relaxation of *NPQ* than *C. fragile* after turning off the actinic light, suggesting that the mechanism of the photoprotective *NPQ* in *D. tuberosus* is partially different from that in *C. fragile*.

Major differences in photosynthetic machinery between the two algae are the absorption spectral profiles and the pigment compositions. Compared to those in *C. fragile*, in *D. tuberosus*, the two photosystems exhibit smaller absorption in both the green and Chl *b* regions, while LHCII exhibits smaller absorption in the green region and larger absorption in the Chl *b* region. These observations indicate that *C. fragile* tends to efficiently absorb blue-green light through any photosynthetic machinery. In contrast, *D. tuberosus* absorbs the Chl *b* region stronger than that from *C. fragile*. Blue-green light is the only light available in underwater environments, especially in intertidal zones (Stomp 2007). Considering that LHCII accounts for the majority of all photosynthetic pigment-protein complexes, the absorption profile of LHCII should reflect that of whole cells. *D. tuberosus* has greater absorption of Chl *b* derived from LHCII than *C. fragile*, which appears to be a better filter to protect the photosystems from blue light and dissipate excess energy. Note that our observation revealed the *D. tuberosus* LHCII binds five xanthophylls: cN, Lo, Vx, Lu and Sn. Compared with the case of LHCII in other Bryopsidales, which binds three xanthophylls: cN, Sx and Sn (Seki 2022b; Li 2023), the hydrophobicity of xanthophylls is obviously increased in *D. tuberosus* LHCII. This increase in hydrophobicity may be one of the factors that shorten the lifetime of Chl fluorescence, as in the case of plant LHCII (Ruban and Johonson 2010). While the Chl fluorescence lifetimes of LHCIIs from intertidal Bryopsidales are almost the same as those in plant LHCII (Li 2020; Brotosudarmo 2022), *D. tuberosus* LHCII may have a shorter lifetime, i.e., a higher quenching ability. The unique Q_y_ absorption pattern of *D. tuberosus* LHCII suggests that the Chl energetics are also significantly different. Further structural elucidation is required to evaluate the quenching capacity of LHCII, and more detailed in vivo and in vitro analyses are needed to clarify the mechanism.

### Plausible binding site of Sn in LHCII from *D. tuberosus*

Recent structural works imply that the green light absorption of LHCII from Bryopsidales can be caused by Sx in the L2 site, which possibly arises from its hydrogen bonds between Sx and surrounding residues. LHCII from *D. tuberosus* exhibited green light absorption as did that from other Bryopsidales. Considering that this alga contains Sn as a carbonyl-conjugated xanthophyll, this green light absorption should arise from Sn in LHCII. The Sn/cN ratio in the *D. tuberosus* LHCII trimer was 1.26 ± 0.01 (Table 1). Based on the equal stoichiometry of cN among the three monomers in the trimer, one Sn is estimated to bind to the LHCII monomer as does cN. Common to the reported structures of LHCII from two intertidal species of Bryopsidales (Seki 2022a, Li 2023), the Sn-binding site (L1 site) contained a hydrophobic cavity to stabilize Sn which contains a hydrophobic acyl group. Thus, Sn is likely to occupy the L1 site of LHCII in *D. tuberosus*, as is the case in the two reported species. From the pigment analysis, we conclude that the Sn molecule binding to the L1 site of LHCII serves as a green light absorber in *D. tuberosus*, implying that the green light absorption is performed by Sn at L1 site or both Sn at L1 site and Sx at L2 site in other Bryopsis LHCIIs. The binding sites and assignment of green light absorption should be confirmed by 3D structure analyses of LHCII from *D. tuberosus* in further studies.

### Plausible binding site of the Sx in a minor monomeric LHCII

The pigment composition analyses have revealed that fraction IV (PSII and PSI core) contains Sx, whereas fraction V (PSI-LHCI super complex) lacks Sx, indicating the presence of Sx within the PSII. Previous structural studies of freshwater green alga have demonstrated that each monomeric LHCII associated with PSII, CP26 and CP29 in green algal PSII-LHCII supercomplex, contains one cN molecule (Shen 2019; Sheng 2019). Consequently, PSII complexes isolated by SDG appear to possess these two monomeric LHCs. Given that the quantity of Sx is half that of cN (Table 1), it is plausible that either CP26 or CP29 binds one Sx molecule at a distinct binding site. In Bryopsidales, the LHCII trimer binds Sx at the L2 site, implying that the L2 site within the LHCII trimer from *D. tuberosus* has the potential to bind Sx. However, our pigment composition analyses showed that the *D. tuberosus* LHCII trimer binds no Sx but Lu, Lo, and Sn, indicating that the carotenoid binding sites of the LHCII trimer exhibit a preferential affinity with these xanthophylls but less with Sx. Notably, Sx is distinguished as the most polar xanthophyll based on its retention time in HPLC chromatogram (Fig. 6), suggesting that one of the monomeric LHCs may have a binding site with high affinity for Sx. In the case of *C. fragile*, LHCII and both photosystems contain Sx, and notably, the photosystems contain more Sx than *D. tuberosus*. *C. fragile* accumulates almost exclusively Sx, Sn, and cN as xanthophylls, whereas *D. tuberosus* accumulates a variety of xanthophylls, mainly Lu, Lo, Vx, and Sn, and only small amount of Sx. The xanthophyll composition of LHCII closely reflects that of the algal body in both species, indicating that these xanthophylls bind competitively to Lhcb proteins. This consideration also suggests that one of the monomeric LHCs in PSII (CP26 or CP29) may have a specific affinity for Sx as a polar xanthophyll. Further biochemical analysis and a high-resolution structure of the PSII complex from *D. tuberosus* are required to determine the binding site and function of Sx in the monomeric LHC.

### Experimental

#### Sample collection

*D. tuberosus* was mainly collected along with environmental water from Oyakebaru-Ukka (26°09′25.1″N, 127°46′57.0″E, Nanjo, Okinawa, Japan) and was also collected from a waterway beside the watercress (*Nasturtium officinale*) field (26 09 06.3 N, 127 47 51.7 E, Nanjo, Okinawa, Japan). The alga was washed with tap water and the contaminants, such as twigs, dead leaves, insects, larvae, and so on, were carefully removed using tweezers and a paintbrush under the freshwater. *D. tuberosus* was incubated in the filter sterile environmental freshwater (0.22 μm, Stericup-GP, Merck Millipore). The light intensities of the environments were measured using a quantum flux meter (MQ-200, Apogee, Logan, USA).

### PAM-chlorophyll fluorescence

Chlorophyll fluorescence parameters were determined using an Imaging-PAM Maxi fluorometer (Heinz Walz GmbH, Effeltrich, Germany) and associated software (ImagingWin, Heinz Walz GmbH). After collection, *D. tuberosus* was incubated under a dim light for 2 weeks at ambient temperature. *C. fragile* (KU-065; KU-MACC, Kobe, Japan) filamentous forms (i.d. ap. 35 μm) were cultivated as described (Seki 2022b). Prior to the PAM measurement, both algae were immersed in fresh filter-sterilized environmental water in a Petri dish and kept in complete darkness for approximately 1 h. After determining minimum chlorophyll fluorescence (*F*_*o*_) at the lowest measured light intensity, the algae were illuminated using a saturating pulse flash and actinic light of a given intensity to determine maximal chlorophyll fluorescence *(F*_*m*_) in the dark and stationary fluorescence (*F*) and maximal fluorescence (*F’*_*m*_) under actinic light. Minimal fluorescence after actinic illumination (*F’*_*o*_) was calculated using the approximation of Oxborough and Baker (1997). The photosynthetically active light absorptivity (Abs) was also measured with the Imaging-PAM Maxi fluorometer. From these data, photosynthetic parameters were calculated as described in Maxwell and Johnson (2000) and Guadagno et al. (2010). ETR was calculated as follows: *Φ*_PSII_ × PPFD × Abs × 0.5, with assuming that the light energy is equally distributed to PSII and PSI. Light-response curves were generated under actinic light by successively increasing PPFD every 30 s after the first illumination of 20 µmol photons m^− 2^ s^− 1^ light for 5 min. The induction kinetics of the parameters were measured by illuminating the algae with actinic light of given intensities for 15 min, with saturating pulse flash irradiated every 20 s. After recording the induction kinetics, dark relaxation experiments were carried out using an automatic program supplied in the ImagingWin software.

#### Preparation of thylakoid proteins

Thylakoid proteins were isolated from *D. tuberosus* and *C. fragile* as described previously (Seki 2022b) with a few modifications: Thylakoid membranes (0.5 mg Chl *a* + *b* mL^–1^) were solubilized with 1% (w/v) of *n*-dodecyl-β-*D*-maltopylanoside (β-DDM) in 50 mM MES-NaOH buffer pH 6.5 containing 20 mM NaCl and 10 mM CaCl_2,_ followed by incubation on ice for 10 min. The supernatant after centrifugation (14,100 g, 5 min, Mini spin plus, Eppendorf Inc.) was loaded onto the top of sucrose layers (0.1-1.0 M stepwise sucrose in the same buffer including 0.03% (w/v) β-DDM). Each fraction after ultracentrifugation was collected and subjected to several rounds of ultrafiltration (Amicon Ultra-15, 50 kDa, Merck) to remove sucrose.

#### Absorption spectroscopy

Each sample solution was poured into a quartz cuvette with an optical path length of 1 cm (Semi micro black quartz cell, T-9 M-UV-10, TOHSO, Tokyo, Japan), and absorption spectrum was recorded using a spectrophotometer (UV-1800, Shimadzu, Kyoto Japan) at ambient temperature. Data was recorded in 0.1 nm increments.

#### Pigment composition analyses

The pigment compositions of the algal body and the thylakoid proteins were determined as described previously (Seki 2022b) with a few modifications: Pigments were extracted from lyophilized algal body as described (Uragami 2014), while pigments were extracted from thylakoid proteins as follows: Thylakoid proteins were adsorbed onto an anion-exchange resin (DE52, Whatman, Cytiva, Tokyo, Japan) packed in a 5-inch Pasteur pipette with a cotton stopper. After lyophilizing the whole Pasteur column, pigments were extracted by a 1:1 (v/v) mixture of methanol and acetone precooled to 4 °C. The extracted solution was dried up by a stream of N_2_ gas and was redissolved into a minimum amount of N, N-dimethylformamide (DMF), and then injected into the UHPLC system at a flow rate of 0.7 ml/min. Pigment compositions were calculated as described (Seki 2022b).

## Electronic supplementary material

Below is the link to the electronic supplementary material.

Supplementary Material 1

## Data Availability

No datasets were generated or analysed during the current study.
