# Supplementary material for: Photosynthetic capacity and pigment distribution of a siphonous green alga, Dichotomosiphon tuberosus
Source: Photosynth Res. 2025 May 21;163(3):30. doi: 10.1007/s11120-025-01148-3 (PMC12095453; doi:10.1007/s11120-025-01148-3)
Supplement: Supplementary file 1 — Supplementary Material 1 [file 11120_2025_1148_MOESM1_ESM.docx]

**Supplementary material** for

**Photosynthetic capacity and pigment distribution of a siphonous green alga, *Dichotomosiphon tuberosus***

Soichiro Seki^1,*,†^, Koichi Kobayashi^2^, Ritsuko Fujii^1,2,3,*^

^1^Graduate School of Science, Osaka City University, ^2^Graduate School of Science, and ^3^Research Center for Artificial Photosynthesis, Osaka Metropolitan University, 3-3-138 Sugimoto, Sumiyoshi-ku, Osaka 558-8585, Japan.

^*^Corresponding authors. E-mail: s-seki@protein.osaka-u.ac.jp (SS) and ritsuko@omu.ac.jp (RF)

^†^Present address: Institute for Protein Research, Osaka University, Suita, Osaka 565-0871, Japan

Table S1

Table S2

Table S3

Table S1. Changes in pigment compositions upon 30 min irradiation at different intensities of white LED/(mmol /mol Chl *b*) ^*1^

|  | Sx^*2^ | cN | Lo | Vx | Ax | Lu | Sn | Chl *a* | β-Car |
| --- | --- | --- | --- | --- | --- | --- | --- | --- | --- |
| Control | 3.4 | 137 | 67 | 223 | n.d.^*3^ | 126 | 79 | 1487 | 90 |
| 300 PPFD | 3.3 | 131 | 66 | 185  (-38)^*4^ | n.d. | 160  (+34) | 78 | 1535  (+48) | 106  (+16) |
| 700 PPFD | 3.4 | 112  (-30) | 77 | 185  (-38) | n.d. | 106  (-20) | 85 | 1439  (-48) | 78 |
| 1000 PPFD | 3.3 | 127 | 75 | 210 | n.d. | 132 | 82 | 1550  (+63) | 115  (+25) |

^*1^ Values are based on one experiment. ^*2^ Abbreviations follow Figure 6 in the main text. ^*3^ Not detected. ^*4^ Differences (> 0.01) from the control are shown in parentheses.

Table S2. Pigment composition relative to Chl *b* (mmol/mol Chl *b*) ^*1^

|  | Sx^*2^ | cN | Lo | Vx | Lu | Sn | Chl *a* | α-Car | β-Car |
| --- | --- | --- | --- | --- | --- | --- | --- | --- | --- |
| Algal body | 10 ± 1 | 130 ± 2 | 107 ± 10 | 237 ± 3 | 94 ± 2 | 91 ± 1 | 1583 ± 15 | 19 ± 0 | 134 ± 3 |
| Fraction III | 2 ± 0 | 69 ± 1 | 81 ± 1 | 132 ± 3 | 76 ± 2 | 87 ± 3 | 595 ± 5 | n.d.^*3^ | n.d. |
| Fraction IV | 38 ± 0 | 88 ± 14 | 56 ± 4 | 100 ± 0 | 50 ± 2 | 69 ± 1 | 2497 ± 64 | 41 ± 4 | 191 ± 11 |
| Fraction V | 3 ± 0 | 28 ± 0 | 31 ± 1 | 181 ± 2 | 113 ± 2 | 130 ± 3 | 4797 ± 40 | 145 ± 17 | 549 ± 28 |

^*1^ Values and errors indicate means ± standard error (N = 3). ^*2^ Abbreviations follow Figure 6 in the main text. ^*3^ Not detected.

Table S3. Pigment composition of Algal body and complexes from *C. fragile* relative to Chl *b* (mmol/mol Chl *b*) ^*1^

|  | Sx^*2^ | tN | cN | Lo | Vx | Lu | Sn | Chl *a* | β-car | α-car |
| --- | --- | --- | --- | --- | --- | --- | --- | --- | --- | --- |
| Algal body | 196 | 4 | 80 | n.d.^*3^ | 6 | 8 | 143 | 1038 | n.d. | 63 |
| Fraction III | 214 | 5 | 94 | n.d. | 6 | 6 | 131 | 795 | n.d. | 15 |
| Fraction IV | 137 | 4 | 52 | n.d. | 4 | 9 | 137 | 1962 | n.d. | 180 |
| Fraction V | 84 | 0 | 39 | n.d. | 0 | 12 | 220 | 2232 | n.d. | 233 |

^1^ Replication was once (N = 1). ^*2^ Abbreviations follow Figure 6 in the main text and the abbreviation of tN is all-*trans* neoxanthin. ^*3^ Not detected.
